# Developing and implementing a methodology for forecasting of medical doctors in Slovenia

**DOI:** 10.1093/eurpub/ckac131.378

**Published:** 2022-10-25

**Authors:** T Albreht, P Ogrin-Rehberger, B Povž

**Affiliations:** Centre for Health Care, National Institute of Public Health, Ljubljana, Slovenia

## Abstract

**Issue/problem:**

Modelling and forecasting the number of health professionals in Slovenia, in particular medical doctors, and nurses, has been a serious challenge for health policy for decades. No serious methodology has ever been implemented to support this process.

**Description of the problem:**

Methodology was developed with the support of the EU SRSS (Structural Reform Support Service) mechanism and further adapted according to the specifics of the Slovenian health system and of the needs of the MoH. The project took two years and was developed jointly with the relevant stakeholders - MoH, National Health Insurance, Medical and Nursing Chambers, Association of Public providers of health care. It was building on the experiences of Austria and Germany. Methodology included the supply and demand side of retrospective data and forecasting data on the population demographic structure as well as the current numbers of doctors and nurses in training.

**Results:**

We have developed a model for forecasting the needs for medical specialists and we tested it on the example of paediatrics, gynaecology, orthopaedics, general surgery, neurology, infectious diseases, and urology. We calculated the forecasted needed numbers for the year 2035 for each of the specialties, where we also indicated the increases and decreases in demand expected based on the demographic, epidemiological and service use projections.

**Lessons:**

Most importantly, the report was endorsed by all the participating stakeholders in the process. The MoH adopted the methodology as a part of the planning mechanism for prospective and current needs for medical specialists in training. Some flaws in the processes identified together with the inadequacies in the updating of data on health professionals led to the MoH’s recommendations to providers on regularly updating their data in the national health providers’ registry.

**Key messages:**

• Methodology developed in the project was implemented as a part of the requirement for the planning of medical specialists.

• Processes enacted in the project contributed to the improvements in the national health providers registry.

